# Characterizing the progress in traumatic brain injuries research in North Africa: a systematic review

**DOI:** 10.11604/pamj.2023.46.99.33297

**Published:** 2023-12-11

**Authors:** Younes Iderdar, Elmadani Saad, Noureddine Elkhoudri, Amina Ibnlfassi, Mohamed Chahboune

**Affiliations:** 1 Hassan First University of Settat, Higher Institute of Health Sciences, Laboratory of Health Sciences and Technologies, Settat 26000, Morocco,; 2 Hassan First University of Settat, Faculty of Sciences and Techniques, Department of Biology, Settat 26000, Morocco

**Keywords:** Traumatic brain injuries, traumatic, progress, research, North Africa, systematic review

## Abstract

Traumatic brain injury (TBI) represents a major health concern worldwide. Currently, systematic TBI studies in North Africa are lacking. Nevertheless, they are highly needed to ameliorate TBI outcomes and increase survival rates among TBI patients. Through this systematic review, we aimed to characterize the progress in TBI research in North Africa and analyse the literature on TBI in the region in the last two decades. A review of North African articles was performed over 22 years (2000-2021) and the required data were collected using keywords: “traumatic brain injury”, “traumatic brain damage”, “traumatic head injury”, and “traumatic head damage”. Abstracts were screened, and selected eligible studies were reviewed independently by two reviewers. The review included 22 studies within the 59,204, 63,083, and 45,918 records that were identified between 2000 and 2021 through Scopus, Web of Science, and PubMed, respectively. The proportion of the total global TBI records that relate to North Africa was less than 1%. Overall, the indices show low progress in the number of new records occurring every year in North Africa and all the records in North Africa were produced after the year 2004. The results show that North Africa has witnessed a low production in TBI research, and the progress is far from being equal to other regions. Production of scientific publications, providing the required information and raising awareness about complications resulting from TBI on individuals and society in general, should be considered.

## Introduction

Traumatic brain injury (TBI) poses a substantial global public health challenge, with projections indicating that its impact results in the annual affliction of more than 10 million individuals, culminating in either fatalities or the need for hospitalization [1]. Roughly 60% of TBIs arise from road traffic incidents across all global regions; approximately 20-30% stem from falls, while 10% result from acts of violence [2]. Numerous years of productive lifespan are lost, and a considerable number of individuals are compelled to endure years of incapacitation subsequent to experiencing a brain injury. Furthermore, this phenomenon incurs noteworthy economic expenditures for individuals, families, and the societal framework.

Research conducted in each part of the world has been vital in understanding the effects of TBI and developing treatments for those affected. Consequently, the implementation of more effective preventive strategies holds the promise of preserving a substantial number of lives and mitigating the onset of years burdened by disability [3].

Within cases of mild TBI, the mortality rate stands at less than 1%; however, for individuals afflicted by a severe TBI, the fatality rate ranges between 20% and 50%. As for the intermediate classification denoted as “moderate” head injury, it is associated with a mortality rate spanning from 2% to 5% [3]. However, even following a mild incident, post-hospitalization disability emerges as a prevalent issue subsequent to TBI [4]. The data pertaining to Tunisia reveals that close to 13,000 individuals experience the repercussions of motor vehicle collisions on an annual basis. Among these cases, approximately 1,500 patients succumb to their injuries, as indicated by statistics from the National Guard [5]. In a study including 437 individuals with TBI in Tunisia, 90% were men with a mean age of 36 ± 17 years and the variables exhibiting a significant association with an unfavorable prognosis encompassed an age surpassing 40 years, a Glasgow Coma Score below 7, the presence of diabetes insipidus, and a blood sugar level exceeding 10 mmol/L [5].

In Morocco, in 2006, 450 head trauma victims over 20 months were admitted in IBN ROCHD University Hospital in Casablanca, with an average age of 38 years; the main cause was road traffic accidents with a percentage of 72.5% and the evolution was favourable in 72.86% of cases with a mortality rate of 25.55% [6]. Since road traffic accidents are responsible for about 85% of the deaths in low- and middle-income countries and constitute the major cause of TBIs, and as North Africa faces a rapid economic transition with increasing motorization and urbanization, TBIs are becoming a leading cause of mortality and morbidity [7]. Furthermore, there is a projection that fatalities resulting from road traffic accidents will undergo an augmentation, transitioning from 1.2 million instances recorded in 2002 to an estimated 1.9 million cases by the year 2030. This escalation is principally attributed to the heightened occurrence of motor vehicle-related fatalities, a phenomenon intrinsically linked to the economic growth being experienced in nations characterized by lower and middle-income countries [8]. By the year 2020, it is anticipated that road traffic accidents will advance in the global burden of disease rankings, transitioning from the ninth position to the third position [3].

The aim of the study was to review all the research reports that were done in the region of North Africa over the last two decades, gain a better understanding of the type of studies conducted in the region, describe their characteristics and distribution in the area, describe the evolution of the research production, and evaluate the contribution of the region to the global research efforts. The study's findings will inform policymakers, researchers, and healthcare professionals on regional TBI research trends, knowledge gaps, and potential future directions. The study's methodical approach will guarantee that the findings are evidence-based and rigorous, advancing TBI studies and clinical practice in North Africa.

## Methods

**Study registration and search strategy:** the systematic review was conducted according to our registered protocol (PROSPERO CRD42023410179). The design complies with the suggestions provided in the “Cochrane Handbook for Systematic Reviews and Meta-Analysis,” with reporting based on the Preferred Reporting Items for Systematic Reviews and Meta-Analyses (PRISMA) guidelines [9].

**Data sources and search strategy:** we established and executed a set of three indices to gauge the advancement of TBI research in the North African region (Table 1 and Table 1.1 show the search strategy for the academic databases). These indices align with the quantity of publications or data extracted from an exploration of three distinct data repositories. The screening process was confined to materials available in the English, French, and Arabic languages. Relevant primary terms were specifically chosen within the search strategy, including “traumatic brain injury” or “traumatic brain damage”, “traumatic head injury”, “traumatic head damage” and “North Africa” or “Morocco”, “Algeria”, “Egypt”, “Tunisia”, “Libya”, “Mauritania”. Both the singular and the plural form of TBI key terms were searched for in the article title, abstract and key words. The indices were as follows: 1) Scopus and Web of Science: the quantity of entries recognized through a comprehensive investigation of research on TBI within the North African context was determined for each database. This exploration involved a strategy incorporating unstructured text and structured field codes, with no constraints related to language or publication year; 2) MEDLINE through PubMed: the entries ascertained via a systematic inquiry of research regarding TBI within the North African region using the PubMed database. It encompassed a comprehensive strategy integrating open-text searches, structured field codes, and Medical Subject Headings (MeSH), without restrictions related to language or publication year. ‘brain injuries, traumatic´ [MeSH Terms] and 'Africa, Northern' [MeSH Terms] were MeSH headings that were exploded to cover all the subheadings.

**Table 1:** the included articles in the review across countries in North African region

Author, year	Country	Sample size	Study design	Objective of study
Youssef M *et al*., 2015	Egypt	67 paediatric patients with TBI	Hospital-based prospective	Ascertain the origins of paediatric TBI while simultaneously monitoring and assessing selected clinical and laboratory indicators to predict outcomes.
Sharf M *et al*., 2013	Egypt	44 adult patients with severe TBI	Observational	Establish a correlation between jugular venous oxygen saturation, GCS, and subsequent outcomes in terms of both morbidity and mortality.
Shehab H *et al*., 2010	Egypt	70 adult patients with closed TBI	Prospective double-blinded	Assess the diagnostic and prognostic efficacy of serum levels of three neuromarkers in patients exhibiting potential signs of intracranial hematoma, in comparison to cranial CT scan results.
Bahloul M *et al*., 2004	Tunisia	437 adult patients with TBI	Retrospective	Examine the frequency and origins of TBI, and identify factors predictive of mortality following TBI occurrence.
Hasanin A *et al*., 2016	Egypt	50 adult patients with severe TBI patients	Prospective observational	Ascertain the occurrence rate of cardiac injury and its associated mortality in individuals experiencing severe TBI.
Fouad H *et al*., 2014	Egypt	46 paediatric patients with TBI	Hospital-based case-control prospective	Examine the utility of D-dimer as a prognostic indicator in children with TBI who are admitted to the Intensive Care Unit.
Fekih Hassen A *et al*., 2012	Tunisia	298 children with TBI	Longitudinal retrospective	Outline the demographic and epidemiological attributes of paediatric TBI.
Bahloul M *et al*., 2009	Tunisia	454 children with TBI	Retrospective	Investigate the epidemiology, etiological factors, clinical presentations, paraclinical manifestations, and outcomes in paediatric patients with TBI.
Mohamed W *et al*., 2018	Egypt	60 adults with severe TBI	Prospective, quasi-experimental	Conduct a comparative analysis of the efficacy between clinical pathway-directed care and conventional care in relation to outcomes associated with hospitalization.
Chelly H *et al*., 2017	Tunisia	694 patients (children and adult) with TBI patients	Retrospective	Assess the post-TBI outcomes and identify predictive factors linked to unfavourable prognoses.
Belatar B *et al*., 2018	Morocco	64 adult comatose patients with severe TBI	Case-control	Examine the potential contribution of heavy metals and trace element imbalances to mortality in patients afflicted by severe TBI.
Ibrahim A *et al*., 2018	Egypt	80 adults with TBI on mechanical ventilation	Prospective observational	Assess the precision of both the semi-quantitative cough strength score and the GCS in prognosticating extubation outcomes among TBI patients.
Ghonemi M *et al*., 2013	Egypt	30 adult patients with isolated TBI	Prospective	Examine the potential diagnostic utility of pNF-H as an early indicator of axonal injury. Furthermore, investigate the potential correlations between pNF-H levels and various clinical variables.
Bahloul M *et al*., 2011	Tunisia	454 children with TBI	Retrospective	Identify the factors linked to adverse outcomes in children who experience traumatic brain injury TBI.

TBI: traumatic brain injury; GCS: Glascow coma scale; CT: computed tomography; pNF-H: phosphorylated neurofilament H

**Table 1.1:** the included articles in the review across countries in North African region

Author, year	Country	Sample size	Study design	Objective of study
Bahloul M *et al*., 2009	Tunisia	222 children with severe TBI	Retrospective	Identify the prognostic indicators associated with mortality among paediatric patients following traumatic brain injury TBI.
Nejmi H *et al*., 2014	Morocco	225 adult patients with moderate or severe TBI	Retrospective	Conduct an evaluation and comparative analysis of the predictive capabilities for in-hospital mortality between the Acute Physiology and Chronic Health Evaluation-II and the Simplified Acute Physiology Score-II specifically for traumatic brain injury TBI.
El Shehaby D *et al*., 2020	Egypt	1221 cases of maxillofacial fractures combined with closed TBI	Retrospective descriptive hospital-based	Examine the legal and medical dimensions of cases involving maxillofacial fractures alongside closed head injuries. Additionally, analyse the demographic characteristics and patterns of various types of such fractures.
Fourtassi M *et al*., 2011	Morocco	42 adult patients with mild TBI	Retrospective	Describe the post-concussion symptoms experienced by Moroccan individuals with mild TBI and investigate the potential connections between persistent post-concussion symptoms and specific dimensions of social and vocational aspects.
Hamdi E *et al*., 2012	Egypt	32 (children and adult) patients with TBI	Prospective	Examine the prognostic significance of cardiac troponin I in patients with TBI.
Belatar B *et al*., 2018	Morocco	64 adult comatose patients with severe TBI	Case-control	Examine the correlation between serum selenium levels and the progression of comatose patients suffering from severe TBI during the initial week of hospitalization. Additionally, investigate the potential association between selenium levels and C-reactive protein.
Bahloul M *et al*., 2011	Tunisia	276 children with isolated TBI	Retrospective	Identify the factors that can predict mortality in children following isolated TBI.
Bensalah M *et al*., 2020	Algeria	133 adult patients with moderate to severe TBI	Prospective	Examine the frequency of post-traumatic hypopituitarism and growth hormone deficiency, and establish their potential relationship with quality of life measures.

TBI: traumatic brain injury; GCS: Glascow coma scale; CT: computed tomography; pNF-H: phosphorylated neurofilament H

The cut-off date for our search was January 2022. In order to search for North African studies, we included country affiliation restriction in all the three databases to obtain studies done by the researchers of the North African countries. In the first step, we obtained a total number of 168.205 articles that concerned TBI all over the world in all the three academic databases. In the second step, we limited the search to North African countries by adding the name of the country to the search key terms to obtain its results. After applying the search criteria, we obtained the results indicated in Table 1 and Table 1.1 that shows the distribution of articles in North Africa and on a global level according to all three academic databases.

**Inclusion and exclusion criteria:** inclusion criteria comprised studies that (1) investigated patients with an initial diagnosis of TBI upon admission, (2) were published between 2000 and 2021, and (3) were cross-sectional, case-control, prospective, retrospective, and clinical trial studies in North Africa. The region refers to the following countries: Algeria, Egypt, Libya, Morocco, Tunisia, and Mauritania. Reports were excluded if the article was only abstract, conference lecture, case report, letter, non-human, non-TBI, conducted outside North Africa as defined above.

**Selection and article screening:** after removing duplicates using Mendeley software, article titles and abstracts were screened using Rayyan web-based software [10] by two independent investigators (Y.I. and N.E.). In the second step, the reviewers (Y.I. and N.E.) retrieved the full texts of articles that met the study´s eligibility criteria independently and screened the full texts of the articles identified. In cases of disagreement, a reviewer (M.C.) was consulted to resolve the disparity.

**Quality assessment:** two reviewers (E.S. and A.I.) evaluated the quality of articles based on the Methodological Evaluation of Observational Research (MORE) as an approach to assess the bias and the quality of the eligible studies.

**Data abstraction and analysis:** the Methodological Evaluation of Observational Research (MORE) checklist was selected as an approach to assess the bias and the quality of the eligible studies. We retrieved all studies meeting the eligibility criteria. To evaluate the severity rates of sustained TBIs, as well as the distribution of TBI literature across North Africa, descriptive analysis of the retained articles was carried out. Microsoft Excel was used to tabulate and display results.

## Results

**Figure 1:**
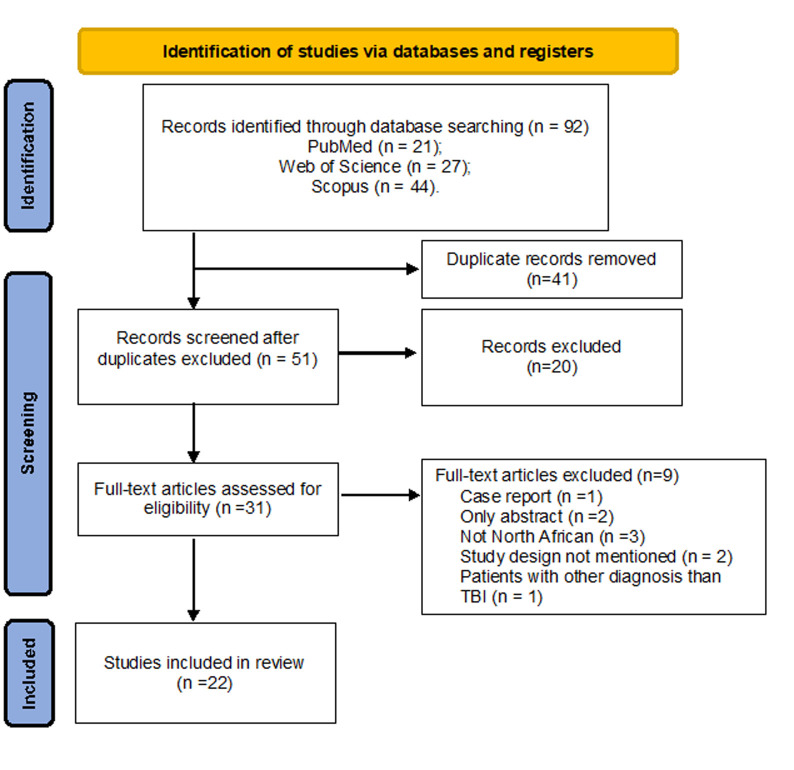
PRISMA chart design for TBIs in the North African region; the PRISMA chart was designed to quantify the progress of TBI research in the North African region; TBI, traumatic brain injury; PRISMA, Preferred Reporting Items for Systematic Reviews and Meta-Analyses

The pursuit of the search strategy yielded a total of 92 articles. Following the removal of duplicate entries and the initial screening process, a final selection of 31 articles aligned with the study criteria. However, within this subset, nine articles were subsequently excluded for various reasons, encompassing instances where the content was only abstracts, case reports, were not conducted on the North African population, study design was not mentioned, and if they included patients with other diagnosis than TBI. A total of 22 full-text articles and reports were included in the final review (Figure 1).

**Figure 2:**
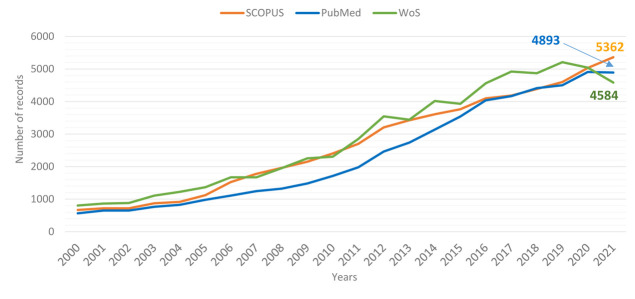
evolution of number of published traumatic brain injury reports in the three databases over the period 2000-2021

The results in Table 2 show differences in terms of the number of articles published at each level. To quantify the contribution of Morocco and North Africa in relation to TBI research worldwide, we employed the three indices. This allowed us to juxtapose North Africa's research output on TBI with the overall global output (Table 2). Since 2000, we identified a total number of 59.204 records through the Scopus Index, 63.083 records through the Web of Science Index, and 45.918 records through the PubMed Index (the distribution of these indices can be seen in Figure 2).

**Table 2:** complete search strategy for the academic databases

Set	Strategy	Results
#1	(“Traumatic brain injury” or “traumatic brain injuries” or “traumatic brain damage” or “traumatic brain damages” or “head injury” or “head injuries” or “traumatic head damage” or “traumatic head damages”)	Scopus: 59.204
		Web of Science: 63.083
		PubMed: 45.918
#2	(“North Africa” or “Northern Africa” or Morocco or Algeria or Egypt or Libya or Tunisia or Mauritania)	
	#1 and #2	Scopus: 57
		Web of Science: 33
		PubMed: 29

**Figure 3:**
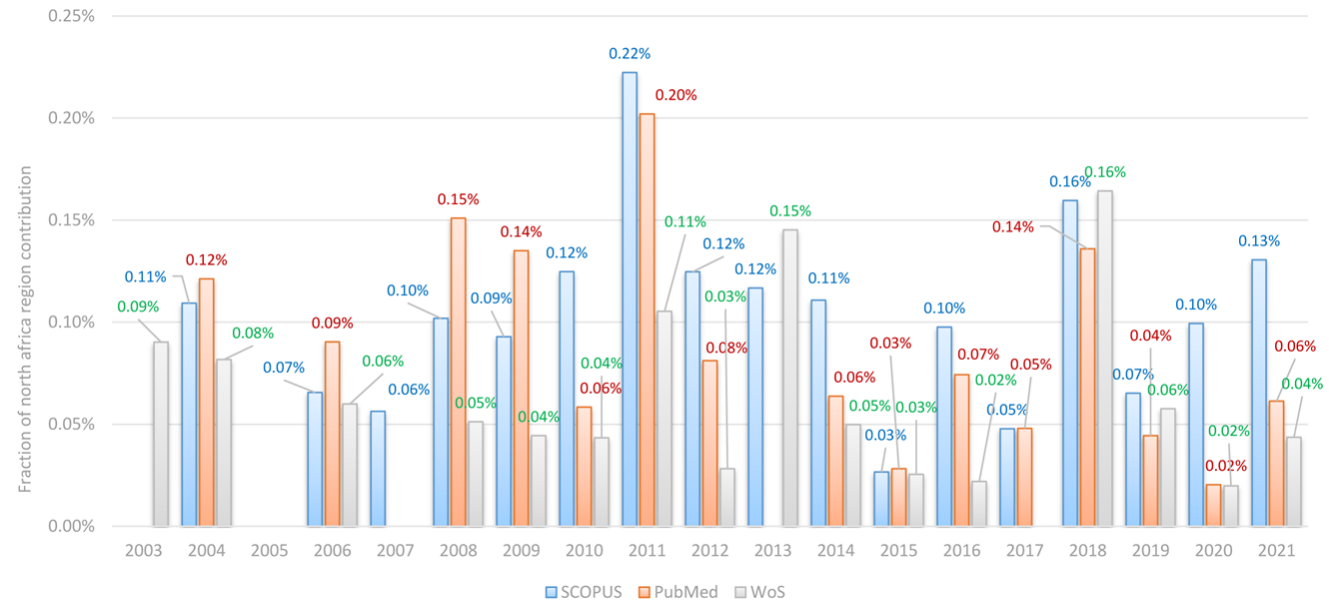
evolution of the fraction of North African traumatic brain injury records relative to global records in all databases over the period 2000-2021

Altogether, the indices illustrate a consistent year-on-year rise in the quantity of newly generated records, but the fraction of North African records in all the databases, relative to global TBI records, showed insignificant levels with a total contribution of less than 1% (Figure 3). Records on TBI started to appear in 1891, but in North Africa, they did not start to appear until 2004 and until 2011 for Morocco, with a very slow rate of progress. There is no evidence so far that there is no research in that field, and yet, there is no evidence of any ongoing research or that TBI research continues to be in an expansion phase in North Africa (Figure 3).

Between 2000 and 2021, the distribution of TBI records through Scopus Index shows differences between the countries of North Africa in terms of the number of published articles. Algeria, for example, has only three articles in Scopus database, seven articles for Morocco, 14 articles for Tunisia, 15 articles for Egypt, and no published articles for Libya and Mauritania.

**Traumatic brain injury study characteristics:** of the 22 studies included in this study, seven studies were in children [11-17], and 13 studies in adults [5,18-28], and three were on both adults and children [29-31]. Sample sizes across all studies ranged from 30 to 1221 participants in total in the included studies, twelve studies had a sample size of 80 participants or less, and ten studies had a sample size of 133 participants or more. The majority of articles were hospital-based studies with multiple natures of evidence.

**Traumatic brain injury regional distribution:** the studies included in the review were distributed across the North Africa region with Egypt reporting the highest number of TBI reports (45%), followed by Tunisia (31.8%), Morocco (18%), and Algeria (4.5%). The remaining countries, Libya and Mauritania, reported no studies.

**Traumatic brain injury severity:** TBI severity, assessed through the Glasgow Coma Scale, was stratified into three distinct categories: mild, moderate, and severe. Notable variations exist in the distribution of cases across these severity levels. Out of the 22 studies, 13 studies captured heterogeneous TBI populations, which included all injury severities, four studies concentrated on severe TBI, two studies concentrated on moderate to severe TBI, and only one study concentrated on mild TBI.

## Discussion

Traumatic brain injury (TBI) presents a significant societal, clinical, and economic load, affecting both the broader community and individuals, as well as straining healthcare systems. This review marks the inaugural endeavour to assemble studies within the North Africa region, with the purpose of evaluating the trajectory of TBI research advancement. It provides valuable perspectives in recognizing the headway achieved in the current TBI literature specific to North Africa, aiming to inform forthcoming policy approaches and research initiatives geared towards enhancing TBI research endeavours.

The input from North Africa to the broader global TBI literature appears notably modest, falling below 1%. The findings of our investigation underscore that the involvement of North Africa in the worldwide TBI discourse is relatively limited. This conclusion remains steadfast across all indices employed to gauge research advancement.

North Africa is perceived to have failed to address the TBI research as a public health challenge. Nevertheless, this small contribution shows a remarkable slow progress in TBI research in North Africa, and in contrast, this progress has greatly improved all over the world. Nonetheless, given the substantial and escalating global health impact attributed to this concern, an imperative exists to expedite the progress of TBI research. Consequently, the Interagency Common Data Elements Project was instituted in 2008, aiming to facilitate the exchange of data and foster collaborative efforts by standardizing definitions and protocols pertinent to TBI research [32,33].

Because of the large gaps in TBI records, we should push ourselves to look for the reasons that prevented TBI research from being a field of interest and we should remain mindful of the value in acknowledging the attained progress, even if it seems marginal. Taken as a whole, the past decade's contributions in North Africa can be characterized as a tentative yet courageous advancement in the field of TBI research, after a period during which the North African countries were just witnessing the rest of the world accelerating their research. This step, nevertheless, was not exactly uniform across North African countries and it was marked by visible disparities in TBI efforts.

Although certain countries have demonstrated progress, others remain considerably lagging in their determination and capability to undertake TBI research endeavours. Libya and Mauritania for example provided no records in these regards. On the other hand, Tunisia produced four times the records that provided Morocco in terms of TBI research but despite all of that, the North African region is still poor in terms of TBI research production.

Scientific progress is being made in some areas, but the fractions of North African TBI records relative to global TBI records in the three databases, show an important deficiency in the volume of research in North Africa (Figure 2). Recognizing that TBIs predominantly transpire within low and middle-income nations [34], this situation underscores inherent vulnerabilities stemming from various influences. These could include potentially inadequate attention to academic publications related to TBIs, a deficiency in public support, and/or insufficiencies in research resources. However, notable headway has been accomplished in the realm of prevention, evidenced by developments such as airbags, seat restraints, innovative vehicle designs, and the promotion of helmet utilization [35].

Nations that are trailing behind in terms of their research productivity should earnestly intensify their endeavours to amplify research initiatives, with the objective of addressing the silent epidemic of TBI within this specific region. In order to expedite advancement, these countries should enhance their momentum by augmenting educational endeavours in the area of TBI. This can be achieved through expanded provision of training grants, research fellowships, and various other training initiatives [35]. Furthermore, it is imperative to initiate efforts aimed at creating repositories or registries specific to the North African region. These repositories should adhere to standardized methodologies and criteria for systematically collecting and reporting data pertaining to cases of TBI. There is a need for future clinical pathway studies in order to improve the patient experience and the hospitalization-related outcomes [24]. The focus of surveillance systems and research studies should primarily be directed towards populations at high risk, as they are the ones most profoundly impacted. Surveys of risk behaviour in the general population and a plan for the future of TBI registries and data systems will be useful to increase awareness of TBI, to encourage research in this field and improve TBI outcomes. Research studies hold significance not only for their immediate advantages but also for their indirect contributions. Allocating funds towards TBI research, clinical efforts, and training initiatives constitute a fraction that falls below 1% of the overall annual expenses associated with TBI [35].

The results of this study showed that the progress of TBI research has been relatively low in the North African region, and requires effective measures and interventions to promote and encourage research in this field. Providing the required information and raising awareness about complications resulting from TBI on individuals and society in general, should be taken into account. Furthermore, comprehending the epidemiological landscape of TBI within the North African region stands as a crucial advancement. This understanding will serve as a pivotal stride towards devising strategic plans and initiatives for TBI prevention and rehabilitation, effective resource allocation, and informing national policies geared towards the reduction of TBI incidences.

**Limitations:** as a limitation in our study, in order to characterize and quantify the progress of TBI research in North Africa, we based the study on the reports and articles published on databases available online; reports that were not published were not included in the review.

## Conclusion

The North African region has observed a notably gradual progression in TBI research, even amid considerable obstacles. Nonetheless, the region's input into the global scholarly discourse remains disproportionately modest in relation to both its population scale and the pressing requirements it faces. Within this context, there exist evident gaps in knowledge that await exploration. Encouraging researchers in different fields and sectors to do more research on TBI will help avoiding a large number of deaths, increasing awareness, and reducing the costs of TBI losses. Furthermore, it is imperative to provide ongoing and sustainable support for research and to ensure the continuity of progress.

### What is known about this topic

- *North Africa is witnessing a large proportion of deaths and disability caused by traumatic brain injuries;*
- *Many years of productive life are lost because of traumatic brain injuries*.

### What this study adds

- *The contribution of North Africa to the global traumatic brain injury research;*
- *The progress of traumatic brain injury research in North Africa*.
